# Therapeutics—how to treat phase separation-associated diseases

**DOI:** 10.1042/ETLS20190176

**Published:** 2020-05-04

**Authors:** Richard John Wheeler

**Affiliations:** 1 Nuffield Department of Medicine, University of Oxford, Oxford OX3 7LF, U.K.; 2 Peter Medawar Building for Pathogen Research, University of Oxford, South Parks Road, Oxford OX1 3SY, U.K.

**Keywords:** cancer, condensates, drug discovery and design, liquid–liquid phase separation, neurodegeneration, therapeutics

## Abstract

Liquid–liquid phase separation has drawn attention as many neurodegeneration or cancer-associated proteins are able to form liquid membraneless compartments (condensates) by liquid–liquid phase separation. Furthermore, there is rapidly growing evidence that disease-associated mutation or post-translational modification of these proteins causes aberrant location, composition or physical properties of the condensates. It is ambiguous whether aberrant condensates are always causative in disease mechanisms, however they are likely promising potential targets for therapeutics. The conceptual framework of liquid–liquid phase separation provides opportunities for novel therapeutic approaches. This review summarises how the extensive recent advances in understanding control of nucleation, growth and composition of condensates by protein post-translational modification has revealed many possibilities for intervention by conventional small molecule enzyme inhibitors. This includes the first proof-of-concept examples. However, understanding membraneless organelle formation as a physical chemistry process also highlights possible physicochemical mechanisms of intervention. There is huge demand for innovation in drug development, especially for challenging diseases of old age including neurodegeneration and cancer. The conceptual framework of liquid–liquid phase separation provides a new paradigm for thinking about modulating protein function and is very different from enzyme lock-and-key or structured binding site concepts and presents new opportunities for innovation.

## Introduction

Liquid–liquid phase separation (LLPS) has emerged as the mechanism underlying the formation of many membraneless organelles (MLOs). This ranges from nuclear domains including nucleoli [1,2] and heterochromatin [3,4], to the formation of stress granules in the cytoplasm [5–7] and formation of signalling and adhesion bodies at the cell membrane [8,9]. Compartments formed by LLPS are termed biomolecular condensates [10,11]. They undergo constant internal rearrangement (reflecting their liquid state), they exist in equilibrium with the surrounding environment (a dilute solvent phase), their formation is reversible and they are distinct from aggregates and protein crystals or polymers [12].

Interest in LLPS has exploded, in part, through the involvement of many condensate-forming proteins in disease, most prominently neurodegenerative disease and cancer [13–15]. Condensates are typically formed by two classes of protein: Firstly, proteins with extensive intrinsically disordered regions (IDRs) termed intrinsically disordered proteins (IDPs) [10,12,16] and secondly, proteins with multiple copies of interaction domains (MCIDPs) [9,17]. High valency transient interactions between either the folded interaction domains of MCIDPs or specialised unstructured regions of particular amino acid composition (called ‘stickers’) in IDPs result in LLPS [10,18]. These proteins are termed the condensate scaffold and are required to form the condensate, while recruitment of client proteins (which cannot themselves undergo LLPS) generates the final condensate composition [19]. However, despite their clear links with key non-communicable diseases, IDPs and MCIDPs are not conventional drug targets.

The conventional view of the druggable genome is the set of protein-coding genes whose product is typical of those that can be modulated by an orally administered small molecule [20], estimated at 10–15% [20–23] of the genome (3000–5000 proteins). This does not typically include IDPs and MCIDPs. A similar proportion of the genome are disease-associated (3961 have entries in OMIM[24]). However, druggability does not correlate well with having a role in disease [20,25]—disease associated IDPs are one example. Our rapidly expanding knowledge of LLPS regulation highlights many druggable proteins which may be valuable targets for conventional drugs. Would it be possible to apply our new understanding of LLPS to make the scaffolds, conventionally undruggable proteins, into new drug targets?

## Why target liquid–liquid phase separation?

A phenotype common in neurodegenerative diseases is aggregation of nuclear or cytoplasmic proteins with IDRs—this includes TAU in Alzheimer's disease and TDP-43 and FUS in amyotrophic lateral sclerosis (ALS) and frontotemporal dementia (FTD). Mutations in these proteins are also associated with more severe pathology [13,26,27]. Neurodegeneration repeat expansion disorders also generate abnormal IDPs including poly-Q in Huntington's disease and poly-FR and poly-PR in ALS/FTD [28–30]. There is evidence for LLPS of all of these proteins, for example TAU canonically stabilises neuron microtubules but can also undergo LLPS [31–33] and concentrate tubulin in the condensate leading to microtubule nucleation [34]. Condensates may also have aberrant properties (different material properties, composition or location of formation) or may more rapidly transition from to an irreversible aggregate when the proteins have disease-associated mutations [7,32,35–37]. Unfortunately, the mechanism of pathogenesis in these diseases is not always clear, for example in ALS only a small proportion of cases involve TDP-43/FUS mutations and, while the mutations are likely causative, it is not completely clear if or how aberrant LLPS is involved [38]. For example, phase-separation deficient TFP43 is retains its splicing activity [39]. Nonetheless, the clear association of LLPS with disease makes these proteins targets of interest.

There is growing evidence for LLPS roles in cancer. Several IDPs associated with neurodegeneration are also oncogenes: EWSR1 (whose fusion with the transcription factor FLI1 causes Ewing's sarcoma) and FUS (whose fusion with the transcription factor CHOP causes myxoid liposarcoma). It is likely, but not proven, that aberrant LLPS of the IDR/transcription factor fusion in the nucleus leads to the activation of tumour-specific enhancers [40]. Another cancer-LLPS link is the tumour suppressor SPOP which interacts with proto-oncogenic substrates and undergoes LLPS, leading to substrate ubiquitination and degradation. Cancer-causing mutations in SPOP prevent LLPS [41]. Molecular detail of these mechanisms are still emerging, but sit as part of wider evidence for likely LLPS-cancer links. Perturbation of any of the membraneless nuclear compartments, whether large (the nucleolus or heterochromatin) or small (transcription factories, DNA damage foci and, in particular, superenhancers [42]) could alter gene expression and contribute to cancer [43–46]. More generally, mechanisms involving LLPS in cell signalling [9,17] can also underlie signalling which, when defective, may contribute to oncogenesis [13]. However overall, the relative importance of defective LLPS as a mechanism in oncogenesis and the diversity of LLPS regulation in different cancers remains to be seen.

Relevance of LLPS is also not limited to non-communicable diseases. Upon viral infection, viroplasm/viral factories often form in the cytoplasm. These MLOs have the liquid properties of condensates for vesicular stomatitis virus and rabies virus [47,48]. Bacteria and fungi can undergo a phase transition of the entire cytoplasm, thought to be protective when stressed or dormant [49,50], and formation of the bacterial nucleoid may be by LLPS [51,52]. Finally, eukaryotic parasites have their own set of vital MLOs with diverse functions which may be formed by LLPS, from the specialised RNA polymerase I transcription factory required for antigenic variation in trypanosomes [53] to the cytoplasmic messenger ribonucleoprotein particles required for expression repression in the female gametes of malaria parasites [54]. These may also be novel targets for intervention.

Unfortunately, IDRs linked with LLPS are a classic example of a canonically undruggable protein domain [55–61], while the protein–protein interaction domains of MCIDs (for example SUMO) are challenging targets [62,63]. IDRs are common in human proteins, half of all human proteins are 20% IDR and 10% of human proteins are more than 50% IDR [64]. Targeting enzymes which control LLPS is therefore the most plausible approach. Furthermore, disease-associated LLPS is a powerful phenotypic readout for high throughput screening [65,66]. However, direct targeting of IDPs/MCIDPs with drugs would be extremely valuable.

## Targeting liquid–liquid phase separations

Targeting LLPS certainly poses challenges, however the conceptual framework of LLPS provides concrete examples and predictions for drug development. This includes both (1) ‘conventional’ drugs which target globular proteins involved in LLPS regulation by signalling or protein post-translational modifications (PTMs) and (2) ‘unconventional’ drugs which directly target the interaction domains leading to LLPS or the physical chemistry of the LLPS system.

Despite many major recent advances, it is important to note that LLPS provides a reductionist model for MLO formation. Cells deviate from physical chemistry models as they are not in equilibrium and scaffolds are often subject to PTMs [32,67–70] and energy-dependent rearrangement by helicases/chaperones [71]. Condensate scaffold are also not uniform polymers and condensates can also contain a huge number of different proteins, unlike typical physical chemistry models. The precise nature of some MLOs is also a subject of debate (whether they are liquid, gel or glass-like) and whether this reflects different phases or a continuum of viscosity.

### The conventional: enzymatic inhibitors

**Figure 1.**
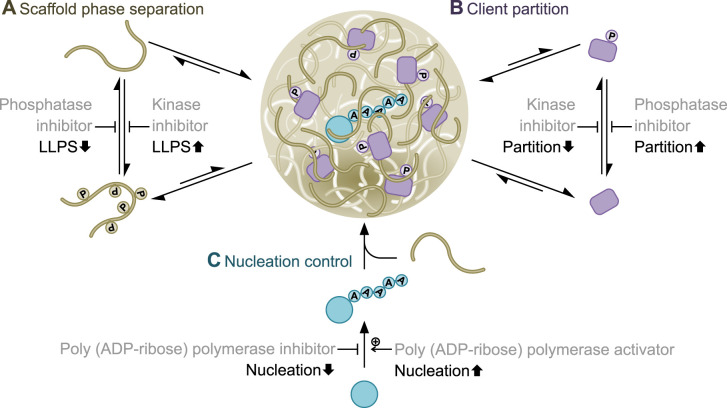
Possible protein liquid–liquid phase separation intervention via post-translational modifications. Cartoon representation of a hypothetical LLPS system, modelled loosely on FUS and TDP-43 [7,67–69,81,89–91,100]. There are several potential points of intervention. (**A**) Phosphorylation (P) of the scaffold (coloured tan) reduces phase separation, therefore a kinase inhibitor would reduce LLPS and a phosphatase inhibitor promote LLPS. (**B**) Phosphorylation of a client (coloured purple) promotes partition to the condensate, therefore a kinase inhibitor would reduce client partition to the condensate and a phosphatase inhibitor would do the inverse. (**C**) Polyadenylation (A) of a key regulatory protein (coloured cyan) nucleates this condensate, therefore a poly(A) polymerase would promote nucleation while an inhibitor would do the inverse.

Scaffold proteins are subject to PTMs which regulate their LLPS [72,73]. Chemical modification of the scaffold changes its physicochemical properties—if this alters it such that LLPS no longer occurs under the current cellular conditions then the condensate will dissolve or *vice versa.* As condensates are an enrichment of scaffold in equilibrium with the surroundings the PTM enzyme could be positioned elsewhere in the cell [74]. Many specific examples of PTM modulating LLPS are known [72,73]: Serine phosphorylation and arginine methylation of FUS both reduce LLPS [67–69] and phosphomimetic mutation of TDP-43 reduces LLPS [70]. In contrast, phosphorylation of TAU [32] and FMRP and CAPRIN1 [75] promotes LLPS and poly-SUMOylation promotes LLPS in SUMO/SIM condensate formation [19]. Aberrant PTMs are associated with disease, making the responsible kinases, phosphatases, methyltransferases, demethylases, SUMO ligases, etc. good therapeutic targets [13,72] (Figure 1A). However, identification of the correct PTM enzyme targets and finding specific small molecule activators and inhibitors will be challenging—diseases, such as ALS, can involve perturbation of many PTM enzymes [76,77]. It is also not clear that PTM interventions will be efficacious until they can be tested in disease models. For example TDP-43 is hyperphosphorylated in aggregates in ALS patients [78,79] despite phosphorylation being implicated in reducing TDP-43 LLPS [70]—evidently phosphorylation has failed to prevent aggregation in late pathology.

Client proteins can partition into condensates and the degree to which they do so depends on the nature of their interactions with the scaffolds and is quantified with a partition coefficient [9,19,80]. PTMs of either the scaffold or client can change the partition coefficient, for example changes to client SUMOylation altering partition to SUMO/SIM condensates [19] and changes to scaffold phosphorylation altering partition of RNA polymerase II to FUS, EWSR1 or TAF15 hydrogels [81]. As control of condensate composition is linked with condensate biochemical function, achieving the correct composition is important [82]. It is therefore also likely there are disease-related PTM enzymes which alter client partition into condensates which would also be conventional targets for small molecule drugs (Figure 1B). However, as above, identifying the relevant PTM enzymes and identifying specific small molecule inhibitors will be challenging.

The kinetics of condensate nucleation are unfavourable, meaning that under conditions where LLPS could occur it may not [12,73]. Mechanisms of nucleation is a complex topic, with only recent advances allowing direct analysis of nucleation [83,84]. Cellular structures implicated in nucleation include membranes, cytoskeleton structures, nucleic acid structures, and specific proteins [71,73,74,80,85–88] but perhaps the best example of a specific molecular mechanism is polyADP ribose (PAR) nucleating TDP-43 and FUS condensates [7,89–91]. PAR polymerases are a conventional target for small molecules and inhibitors reduce TDP-43-associated pathology in *in vitro* neurons, a very promising result demonstrating control of nucleation as a therapeutic target [90]. Another specific molecular example is Nephrin phosphorylation leading to the Nephrin signalling MLO [92]. Together, this points to PTMs often controlling nucleation, and the enzymes responsible would be conventional targets for small molecule drugs [90,91] (Figure 1C). However, nuanced intervention will require understanding of both when and where condensates should form for their normal function.

Finally, whether LLPS occurs is dependent on the concentration of the scaffold. Cells have many ways of regulating protein or nucleic acid level in different compartments, from simply how much has been synthesised, to organelle import/export. This is a more multi-faceted topic, but there are clear further possibilities for targeting by conventional small molecule drugs.

Together, these opportunities from conventional drug targets highlight the value of LLPS as a phenotype for screening existing drug libraries. This is subject to the many advantages and various challenges of efficacy and specificity, perhaps best understood for kinase drugs [93], and any hits would be amenable to conventional analyses of mode of action, analyses of specificity and efficacy, optimisation of leads by medicinal chemistry paradigms—overall potentially highly productive.

### The unconventional: physicochemical mechanisms

The possibility of directly targeting LLPS has gathered great interest [94], particularly through our pre-print describing small molecules which modulate LLPS of ALS-associated stress granule proteins [66]. Perhaps because of a tendency to view ligandability as a strict pre-requisite for druggability [95–98], efforts to target canonically undruggable proteins tend to focus on finding binding sites, whether they are in non-enzymatic structured domains [62], cryptic binding sites [99] or transient binding sites in IDPs [57,61]. However, IDPs and MCIDPs in a condensate are suspected to have large scale conformational unwinding which enhances the capacity to undergo high valency interactions required for LLPS [9,19,100,101]. Recent evidence points to a balance of sticker (sequence sections which tend to cross-interact) and spacer (sections which do not) leading to LLPS without tertiary structures forming [100,102–105], although some studies indicate transient tertiary structures do form [106]. Is it possible to have drug interaction with an IDP without requiring a binding pocket? LLPS suggests it is possible through a physicochemical mechanism of action and this novel approach opens up new concepts for traditionally undruggable cellular targets.

The effects of the aliphatic alcohol 1,6-hexanediol are perhaps the best evidence that physicochemical disruption of LLPS is possible. 1,6-hexanediol was originally used to analyse FG rich IDRs in the nuclear pore complex [107–109] and was later found to disrupt many MLOs [45,110,111]. However, high concentrations (0.1–1%, high mM) are required and it has many aberrant effects [112]. 1,6-hexanediol, like many compounds including alcohols and dimethylsulfoxide (DMSO), alters hydrogen bonding and therefore alters hydrophobic interactions which, in turn, alters LLPS [107–109]. General solvent effects like these are too broad to be of use as a therapeutic. However it is notable that cells maintain high adenosine triphosphate (ATP) concentrations and, like 1,6-hexanediol, ATP reduces stress granule protein LLPS *in vitro* [113] and in cells [114].

There are several other more therapeutically plausible approaches to directly modulate LLPS. First is through polyphasic linkage—the interplay between protein phase and protein–ligand interaction. If protein–ligand binding is preferred when the protein is in a particular phase (for example, preferring the solution over the solid phase) then more ligand binding shifts the system equilibrium to prefer the corresponding protein phase, or vice versa. Examples of polyphasic linkage are known for several solid to solution phases. The classic example is haemoglobin crystallisation dependent on the oxygen ligand concentration [115–117]. In neurodegenerative disease, huntingtin aggregation is dependent on the profilin ligand concentration, via a soluble oligomer intermediate [118]. These concepts should also apply to liquid phases, indeed LLPS of the ALS-associated protein UBQLN2 shows polyphasic linkage with its ligand ubiquitin [119]. Therefore small molecules which preferentially bind a scaffold when either soluble or when in a liquid phase will modulate LLPS and may have therapeutic potential.

Second is through partition of molecules into a condensate altering the condensate properties. This is well characterised for partition of client proteins: The partitioned protein itself confers new properties to the condensate while simultaneously displacing other proteins which partitioned to the condensate through similar client-scaffold interactions [19]. Furthermore, high concentrations of a client protein can destabilise a condensate, likely through entropic effects [10,19,82,120]. This is arguably a form of polyphasic linkage as client binding results in an effective preference for the soluble phase of the scaffold. Small molecules, like proteins, can partition into a dilute or condensate phase. The best evidence comes from non-cellular systems: partition of neutral hydrophobic and aliphatic small molecules into various synthetic polymer condensates [121–123], partition of enzyme substrates into condensates where the enzymes are clients of the condensate [124], partitioning of dye molecules into condensate models of proto-cells [125,126] and partition of small ions out of polyelectrolyte coacervate condensates [127]. The partition of a client depends on several contributing factors to the free energy of the solute (the client) in the different coexisting liquid phases likely dominated by hydrophobic interactions, charge and hydrogen bonding depending on the properties of the scaffold [10,128], in practice this will be dominated by the properties of stickers for IDP scaffolds. A small molecule which partitions to a condensate, even without strong binding, may therefore have therapeutic value.

Thirdly, more complex interactions also seem plausible, in particular interaction with the condensate surface. There is evidence for accumulation of small dye molecules to condensate interfaces and a terpolymer has been used to stabilise a protocell coacervate [126]. Here, part of the molecule partitioned into the condensate and part out in a surfactant-like manner [126] which, like surfactants, may stabilise condensates at low concentrations and destabilise them at higher concentrations.

### The synergistic

It is widely suspected that condensates provide a specialised environment for biochemistry through molecular crowding, scaffold and client biochemistry and local solvent environment [128]. They can promote enzymatic activity inefficient in the cytosol [129], concentrate proteins to promote their interaction [34,80] or sequester proteins to prevent activity [130]. There are further strong predictions from biomolecular chemistry [18,128] and a rapidly growing number of *in vitro* models including promoting actin nucleation [131], enhanced ribozyme activity [132] and enhanced enzyme activity [124,125,133–135]. If disease-associated enzymes are condensate clients then a synergistic approach to drug optimisation could be taken—linking a small molecule inhibitor with a molecule that partitions to the condensate to confer enhanced efficacy and/or specificity.

## Is a physicochemical mechanism plausible?

**Figure 2.**
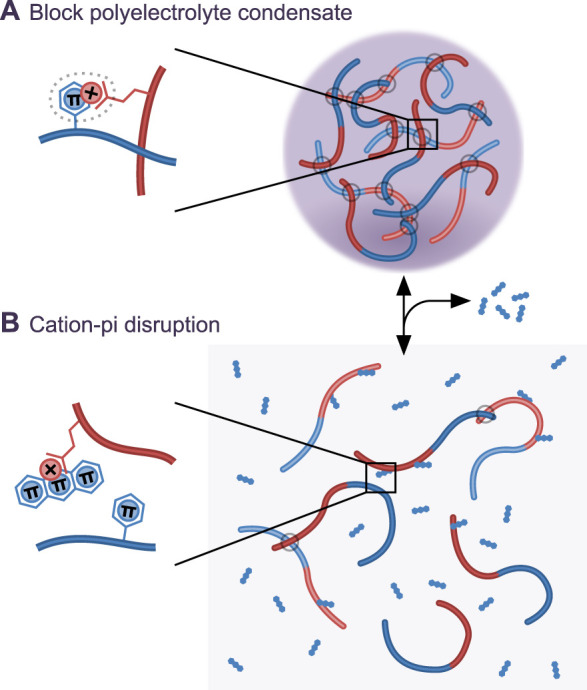
Physicochemical intervention of protein liquid–liquid phase separation?. (**A**) Cartoon representation of a model block polyelectrolyte condensate whose formation involves cation-π interactions inspired by synthetic polymer [136] and FUS-like [100] LLPS. (**B**) A compound which partitions to the condensate and reduces effective strength of cation-π interactions hypothetically leads to condensate disruption.

Existing evidence indicates a physicochemical mechanism is plausible, however existing evidence primarily involves the effect of proteins rather than small molecules on LLPS. Providing evidence for a physicochemical mode of action of a small molecule using normal drug development paradigms is challenging as it does not necessarily require strong small molecule binding to the target. Evidence for target engagement will therefore also pose challenges. However, there is early evidence small molecules can modulate LLPS from synthetic polymers. Methylene blue partitions to a polyelectrolyte coacervate through hydrophobic interactions [123] and reduces the effective strength of π–π and cation-π interactions, modulating whether LLPS or aggregation occurs and changing condensate properties [136]. Planar heterocyclic small molecules including mitoxatrone, similar to methylene blue, have been identified as preventing stress granule protein LLPS [65,66]. It was suggested they act through disrupting RNA base stacking by intercalation [65] however can act on RNA-free *in vitro* LLPS [66] perhaps reducing cation-π interaction required for FUS-like protein LLPS [100] (Figure 2). Our work also identified lipoamide as a stress granule LLPS modulating compound, with physicochemical effects *in vitro* and specific effects on stress granules in cells [66]. This suggests that small molecules that reach high concentration by partition to specific condensates could indeed alter LLPS.

Could this approach be efficacious? A small molecule needs to reach the target, have sufficient specificity and have a beneficial effect. Conventional small molecule therapeutics are guided by Lipinski's rule of five [137] and similar [138,139] to bias towards good oral bioavailability. Will small molecules with a physicochemical mechanism tend to meet these guidelines? And would they be amenable to medicinal chemistry to better meet these criteria? This also assumes that condensate modulation is beneficial to patients—the precise pathomechanisms are unclear in many of these diseases and aberrant condensate formation/dissolution may be a phenotype rather than a cause.

Specificity is an important concern. It is not yet clear that different condensates may be targeted specifically by partition of a small molecule to only the target condensate. 1,6-hexanediol affects many condensates [45,110–112] and sets an unfortunate precedent. On the other hand, coexisting immiscible condensates are common, perhaps best exemplified by the nucleolar sub-compartments [2]. Immiscibility points to different physicochemical scaffold properties giving specific partition of clients and presumably allowing specific partition of a small molecule [74]. Growing knowledge of the molecular grammar of the stickers involved in IDP scaffold LLPS [100,102–105] may even allow guided physicochemical drug design, analogous to structure-based drug design, although perhaps not in the near future. Broader concerns are sensitivity of condensates in target cells (e.g. cancer cells or neurons) compared with off-target cells. Aberrant condensates, such in Ewing's sarcoma [40], may be only present in the target cell, but form by LLPS using scaffolds (in this case, EWSR1) used in all cells for other physiological condensates. Therapeutic possibility here hinges on the side effects of disrupting normal EWSR1 function in balance with the benefits of disrupting a nuclear EWSR1 fusion protein.

The necessary pre-requisite for any high throughput screen is that small molecules with activity exist in the screened library, and this library is normally filtered to avoid small molecules with undesirable physicochemical properties (e.g. aggregation [140]) and small molecules which often interfere with assays (termed PAINs [141]). For a physicochemical mechanism, arguably small molecules viewed as having poor physicochemical properties should be included—small molecules which can form micelles, colloids or other aggregates might be highly effective against the physical chemistry of LLPS and be a true hit, while they would interfere with *in vitro* assays for recombinant enzyme inhibition. Arguably, it may also be desirable to avoid widely successful chemical fragments or privileged scaffolds to avoid interaction globular proteins which would be an off-target effect. Screening will also require novel approaches. Perhaps partition to *in vitro* condensates or phenotypic screens for effects on condensates in cells.

## Conclusions

Many MLOs formed by LLPS are associated with disease through a combination of condensates forming in aberrant locations, with aberrant composition or aberrant physical properties. This suggests many possibilities for therapeutics. PTM enzymes are canonical drug targets and PTM often controls LLPS, condensate composition and condensate nucleation. Furthermore, presence of condensates is a simple phenotypic readout to screen for hits. However, in many neurodegenerative diseases and cancers the pathomechanism is not concrete, is LLPS actually a good target or is aberrant LLPS simply a consequence of pathology? More basic research into these disease pathomechanisms are needed to confer confidence in LLPS as a target.

More speculatively, direct targeting of LLPS may be a novel therapeutic approach. Most drug targets are enzymes, ion channels, G-protein coupled receptors, kinases, nuclear receptors and transporters. However, well over half of the genome, including IDPs, does not fall into these classes [25,142,143]. The physical chemistry of LLPS for condensate formation provides a new conceptual framework for how small molecules may interact with IDPs—one which does not necessarily involve well-defined binding sites and instead physical chemistry mechanisms. However, finding these molecules may need careful library design and re-evaluation of what defines a small molecule as drug-like. It is also not yet unambiguous if physicochemical mechanisms will be efficacious and specific.

## Summary

- Many roles of liquid–liquid phase separation have emerged in neurodegeneration and cancer.
- Aberrant location, composition or physical properties of condensates formed by liquid–liquid phase separation are associated with disease.
- Recent discoveries show how protein post-translational modification can regulate condensate nucleation, growth and composition.
- There is great potential for small molecule inhibitors of post-translational modification to modulate liquid–liquid phase separation.
- More speculatively, direct physicochemical action of small molecules on liquid–liquid phase separation may have therapeutic potential.

## Open Access

Open access for this article was enabled by the participation of University of Oxford in an all-inclusive *Read & Publish* pilot with Portland Press and the Biochemical Society under a transformative agreement with JISC.

## Abbreviations

IDP: intrinsically disordered protein
IDR: intrinsically disordered [protein] region
LLPS: liquid–liquid phase separation
MCIDP: multiple copies of interaction domain protein
MLO: membraneless organelle
PTM: post-translational modification
